# Composition and diversity of bacterial communities in the rhizosphere of the Chinese medicinal herb *Dendrobium*

**DOI:** 10.1186/s12870-021-02893-y

**Published:** 2021-03-04

**Authors:** Jiajia Zuo, Mengting Zu, Lei Liu, Xiaomei Song, Yingdan Yuan

**Affiliations:** 1 College of Horticulture and Plant Protection, Yangzhou University, Yangzhou, 225009 China; 2 Joint International Research Laboratory of Agriculture and Agri-Product Safety, the Ministry of Education of China, Yangzhou University, Yangzhou, 225009 China

**Keywords:** Microbial community, 16 s rRNA, Rhizosphere soil, *Dendrobium*, WGCNA

## Abstract

**Background:**

*Dendrobium* is a precious herbal that belongs to Orchidaceae and is widely used as health care traditional Chinese medicine in Asia. Although orchids are mycorrhizal plants, most research still focuses on endophytes, and there is still large amount unknown about rhizosphere microorganisms. To investigate the rhizosphere microbial community of different *Dendrobium* species during the maturity stage, we used high-throughput sequencing to analyze microbial community in rhizosphere soil during the maturity stage of three kinds of *Dendrobium* species.

**Results:**

In our study, a total of 240,320 sequences and 11,179 OTUs were obtained from these three *Dendrobium* species. According to the analysis of OTU annotation results, different *Dendrobium* rhizosphere soil bacteria include 2 kingdoms, 63 phyla, 72 classes, 159 orders, 309 families, 850 genera and 663 species. Among all sequences, the dominant bacterial phyla (relative abundance > 1%) were Proteobacteria, Actinobacteria, Bacteroidetes, Acidobacteria, Firmicutes, Verrucomicrobia, Planctomycetes, Chloroflexi, and Gemmatimonadetes. And through WGCNA analysis, we found the hub flora was also belong to Acidobacteria, Actinobacteria and Proteobacteria.

**Conclusions:**

We found that the rhizosphere bacterial communities of the three kinds of *Dendrobium* have significant differences, and that the main species of rhizosphere microorganisms of *Dendrobium* are concentrated in the Proteobacteria, Actinobacteria, and Bacteroidetes. Moreover, the smaller the bacterial level, the greater the difference among *Dendrobium* species. These results fill knowledge gaps in the rhizosphere microbial community of *Dendrobium* and provide a theoretical basis for the subsequent mining of microbial functions and the study of biological fertilizers.

**Supplementary Information:**

The online version contains supplementary material available at 10.1186/s12870-021-02893-y.

## Background

Rhizosphere soil refers to the narrow zone of soil affected by root exudations, containing up to 10^11^ microbial cells and over 30,000 prokaryotic species [1–3]. Rhizosphere microbes are significantly different from non-rhizosphere microbes in terms of species, number, and activity [4]. There are abundant microbial resources in rhizosphere soil, which can be 10–1000 times that in bulk soil [5]. The rhizosphere bacterial communities are represented by diverse bacterial taxa, though their abundance varies across the root system under different soil and plant types [6]. In addition, the contents of the Firmicutes and Acidobacteria are also high [7]. Using high-throughput sequencing, Lundberg et al. found that *Arabidopsis thaliana* had the highest relative abundance of Proteobacteria in the rhizosphere bacterial community, with Pseudomonadaceae being the main population, followed by Bacteroides and Actinomyces [8]. The interaction between rhizosphere microbes and plants can affect material circulation and energy flow. Changes in its community structure and abundance can affect plant growth and development, flowering and fruiting, and plant interaction with phytophagous insects, which is of great significance for plant growth and yield [9, 10]. *Dendrobium* spp. is the second largest genus in Orchidaceae, with more than 1400 species in the world. There are 74 species and 2 varieties of *Dendrobium* in Orchidaceae in China, most of which are precious medicinal plants. *Dendrobium* has many functions, such as benefiting stomach and promoting body fluid, clearing heat and nourishing yin, relieving inflammation and relieving pain, clearing eyesight, and enhancing immunity [11]. Currently, artificial cultivation using non-symbiotic tissue culture does not meet market demand because of slow growth and low survival rates [12]. Therefore, the development of an effective method for propagating these endangered species for both conservation and commercial production is needed. There are three kinds of medicinal *Dendrobium* in the Ta-pieh Mountain area, which are *Dendrobium huoshanense*, *Dendrobium officinale* and *Dendrobium moniliforme* [13]. All of them are perennial herbs of *Dendrobium* in Orchidaceae. They contain mainly polysaccharides, alkaloids and bibenzyls. They have antitumor, immunomodulatory, antioxidant, vasodilator and hypoglycemic effects [14].

At present, the research in *Dendrobium* mainly focuses on the endophytic bacteria. Indeed, there is a complex relationship between *Dendrobium* and its endophytes. Previous studies have shown that Sphiugomouas and Mycobacterium bacteria isolated from the roots of *D. moschatum* (Buch. - ham) SW. could significantly improve the seed germination rate of *D. moschatum* (Buch. - ham) SW [15]; and there are many studies using protocorm as material to successfully isolate and obtain effective fungi that promote seed germination [16–18]. However, few reports are available regarding *Dendrobium* and soil microbial communities. There are abundant bacteria, fungi and actinomycetes in the rhizosphere of medicinal plants. Not only can they promote the absorption of soil nutrients by medicinal plants, but they can also improve the yield and quality of medicinal plants; however, they can also cause continuous cropping obstacles for medicinal plants. So, studying *Dendrobium* medicinal plants and their rhizosphere microbes is of great significance to clarify how rhizosphere microbes can improve the yield and quality of medicinal plants. However, the traditional cultivation methods account for 0.1 to 1% of the environmental microorganisms, which cannot fully reflect the real situation of the environmental microbial community. In recent years, with the rapid development of new generation sequencing technology, based on 16 s rRNA sequence amplification and Illumina Miseq high-throughput sequencing technology, huge amounts of data can be obtained. By means of bioinformatics, the overall microbial community composition can be obtained. With the advantages of high-throughput, low price and short operation cycle, it has been widely used in the study of microbial community structure. Especially in the field of medicinal plants, such as *Panax ginseng* [19], *Panax notoginseng* [20], *Ajuga bracteosa* [21], *Origanum vulgare* [22], *Lilium davidii* [23] and other plants,these methods are widely used.

They always interact with other microorganisms such as mutualism, parasitism, antagonism. Co-culture is often used to study the interaction between microorganisms [24], but due to the inculcability of most microorganisms, co-culture has shown great limitations. Therefore, it is necessary to find other reliable ways to study the interaction between microbial communities. The rapid development of high-throughput sequencing has provided a new method for the study of microbial interaction. Based on high-throughput sequencing results, researchers have developed a number of software to predict the interaction among microbial communities, such as CoNet, MENA [25]. These network analyses reflect the co-expression relationship among microbial communities. Weighted gene co-expression network analysis (WGCNA) is a kind of gene expression can be measured with a specific character combined with the analysis of network analysis algorithm [26]. It can divide the gene co-expression network of complex biological processes into several highly correlated characteristic modules, and can establish the correlation between the modules and specific traits, so as to find the hub flora. WGCNA analysis has been widely used in transcriptional data analysis of various plants (such as corn [27], tomato [28] and *Dioscorea nipponica* [29]). But now, WGCNA analysis also has many applications in microbiome, such as rhizosphere microbes of potatoes [30].

Therefore, the goal of our work reported here was to characterize the rhizosphere microbial community of different *Dendrobium* species during the maturity stage. Our specific objective was to describe taxa associated with each *Dendrobium* species and determine which the environmental factors were related to microbial diversity and community composition. Specifically, we hypothesized that: (1) the dominant genus bacteria of *Dendrobium* and its comparison with other medicinal plants; (2) rhizosphere community composition will differ between three different *Dendrobium*; and (3) the hub flora can be obtained through WGCNA analysis.

## Materials and methods

### Plant material and soil sampling

*Dendrobium* plants were artificially cultivated in the greenhouse of Anhui Tongjisheng Biotechnology Company, Lu’an, China. The original source was collected by the company from the wild after obtaining local permission. The protocols and the conditions of planting for the growth of protocorm-like bodies were described by our previous study [31]. From the grown plants of the three species of *Dendrobium*, two-year-old *D. huoshanense*, *D. moniliforme* and *D. officinale* were selected to provide eight replicates of each sample. The voucher specimens were authenticated by Professor Maoyun Yu from West Anhui University and deposited at Jiangsu Key Laboratory of Crop Genetics and Physiology in Yangzhou University, Yangzhou, China (Voucher number: 20A01, 20A02 and 20A03).

To obtain rhizosphere soil, plants were removed from flowerpots and large soil aggregates were removed by hand; soil firmly attached to roots was collected with sterile brushes and regarded as rhizosphere soil. The rhizosphere soil was sampled and sieved to remove plant debris. A part of the soil samples was put into sterile centrifuge tubes, frozen in liquid nitrogen immediately, and then stored at − 80 °C until the soil microbial composition was analyzed.

### DNA extraction, PCR amplification and 16S rRNA sequencing

For each flowerpot we obtained a single DNA sample, which was used for 16S rRNA sequencing. Total DNA extraction and concentration were used DNeasy Power Soil Kit (Qiagen, Valencia, CA, USA), Qubit® dsDNA Assay Kit (Life Technologies, CA, USA) and DNA degradation degree and potential contamination were monitored on 1% agarose gels [32].

For bacterial diversity analysis, V4 hypervariable regions of 16S rRNA genes were amplified with universal primers 515 F (5′-GTGCCAGCMGCCGCGG-3′) and 806 R (5′-GGACTACHVGGGTWTCTAAT-3′) [33], and then PCR products were sequenced by IonS5™XL sequencing technique platform [34]. Quality filtering on the raw reads were performed under specific filtering conditions to obtain the high-quality clean reads according to the Cutadapt (V1.9.1) [35] quality-controlled process. Chimeric sequences were removed by comparing with the Silva database [36] using UCHIME algorithm [37] to detect chimera sequences, and then the chimera sequences were removed. Operational taxonomic units (OTUs, cutoff 97% sequence identity for 16S rRNA) were clustered by UPARSE software (v7.0.1001) [38]. The phylogenetic taxonomy was assigned according to the Ribosomal Database Project (RDP) classifier at an 80% confidence threshold (Version 2.2) [39] using the Silva databases for bacteria. Alpha-diversity was described for each sample using the following metrics for the observed species (OTU numbers); Chao1, Shannon and Simpson index, ACE and Good-coverage, which were generated to compare the level of bacterial OTU diversity [40].

### WGCNA analysis

#### Construction of weighted OTUs co-expression networks and identification of modules

The co-expression network of OTU was constructed using the WGCNA program package in R software [41]. Co-expression module construction was according to Wan et al. methods [42]. We used the function (pickSoftThreshold) to calculate the appropriate weighting coefficient β. The selection of the soft threshold should satisfy that the square of the correlation coefficient is close to 0.8 while ensuring that the connectivity cannot be too low. The WGCNA algorithm was used to construct the modules to extract the OTU information corresponding to each module after the soft threshold is determined.

#### Interactions analysis of co-expression modules

According to the soft threshold selected above, the adjacency matrix was calculated, and the topological overlap matrix (TOM) was further constructed. The value in the TOM matrix reflects the similarity of the co-expression relationship between two OTUs. The higher the similarity, the closer the value is to 1. Then, we used the function (flashClust) to perform hierarchical clustering and used the dynamic cut tree algorithm (minModuleSize = 25) to cut the gene cluster tree for module division [41]. At the same time, colors were randomly assigned to each module, and principal component analysis was used to calculate the module feature vector of each module. The module feature vector was used to estimate the relationship between the module and the trait, the samples were classified according to the corresponding characteristics, and the module with the correlation coefficient ≥ |0.5| and *p* value ≤0.01 was selected for further analysis.

#### Identification of hub OTUs

Hub OTUs are often used as an abbreviation for highly connected OTU, which has a high degree of connectivity in the co-expression module. In our research, according to the size of the module, we classified the top 20 OTUs with the highest correlation as hub OTUs. At the same time, we used Cytoscape (v.3.6.1) to build and visualize the interaction network between OTU and OTU [43].

### Statistical analysis

The WGCNA software package, stat and ggplot2 packages in R software (Version 2.15.3) were used for PCoA and WGCNA analysis. We conducted the co-occurrence network analysis in different rhizosphere soil compartments. The network analyses were performed using the psych R package (v.1.8.12) [44]. The bipartite network was constructed to feature the shared phyla among different samples [45]. The bipartite network was visualized using Cytoscape (v.3.6.1) [43].

## Results

### α- and β-diversity of soil microbial communities

**Fig. 1:**
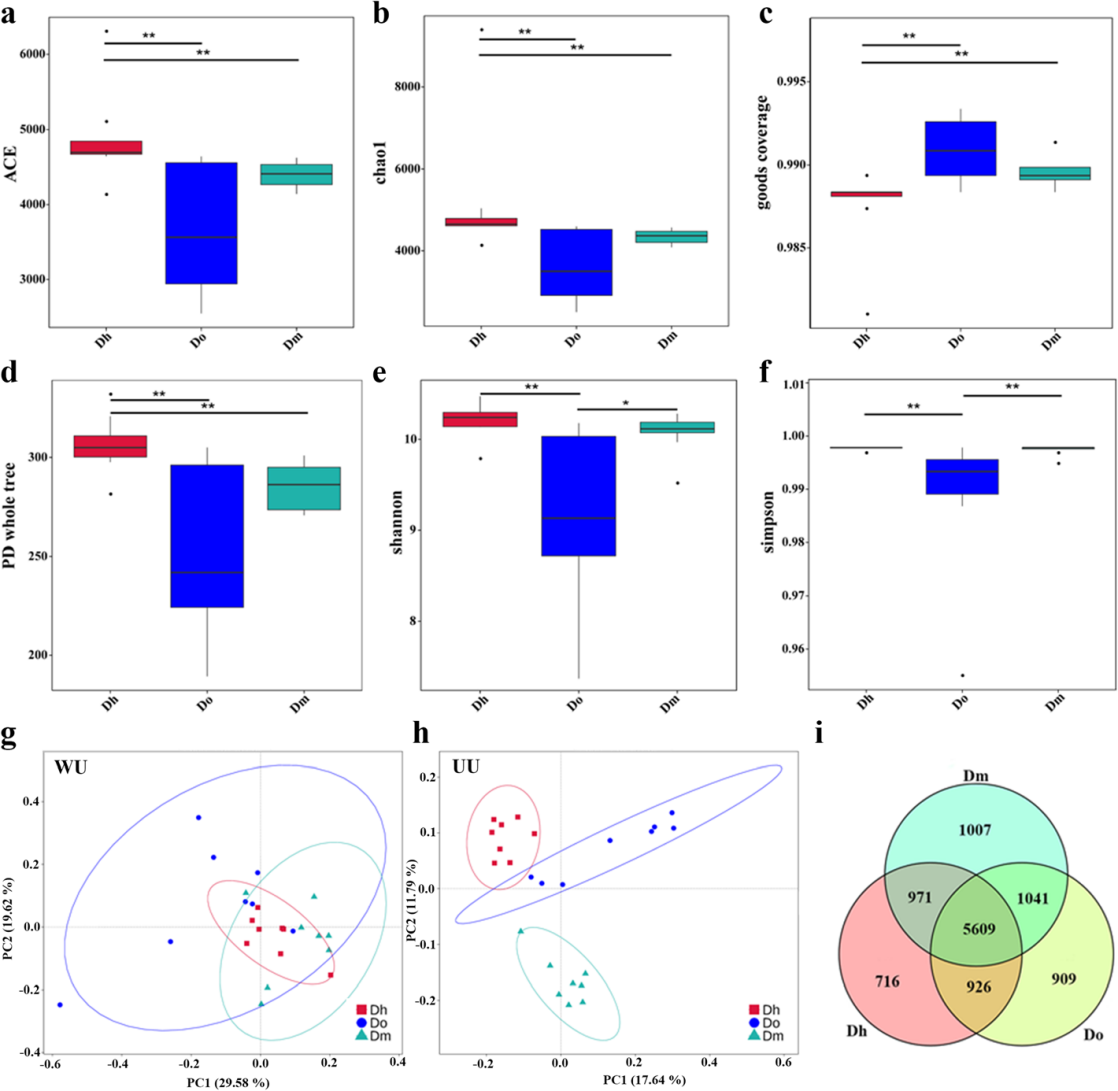
**a-f** α-diversity of three *Dendrobium* species: **a**. ACE; **b**. chao1; **c**. goods coverage; **d**. PD whole tree; **e**. shannon; **f**. simpson. **h-i** PCoA analysis of β-diversity based on the weighted UniFrac (WU) and unweighted UniFrac (UU) distances: **h**. WU distance; **i**. UU distance. Symbols with different colors represent different species: red square: *Dendrobium huoshanense*; blue circle: *Dendrobium officinale*; green triangle: *Dendrobium moniliforme*. **j** Venn diagram representing bacterial operational taxonomic units (OTUs) associated with the rhizosphere of *Dendrobium*

The rhizosphere soil alpha diversity indexes of different *Dendrobium* species are different (Fig. 1). The Good’s coverage index of *Dendrobium* library was greater than 98.5%, indicating that the sequencing results reflect the real situation of the bacterial population in the sample. We found that *Dendrobium huoshanense* (Dh) had the highest Shannon diversity index (10.29), while *Dendrobium officinale* (Do) had the lowest (9.16), which indicated that Dh rhizosphere soil has the highest diversity of bacterial communities. The index of ACE and Chao1 have the same trend, Dh is the highest, respectively 5225 and 4877, indicating that Dh samples have the highest community richness. Further analysis revealed that the Shannon index and Simpson index of soil bacteria of Dh was significantly different from Do, and Do was significantly different from *Dendrobium moniliforme* (Dm).

**Fig. 2:**
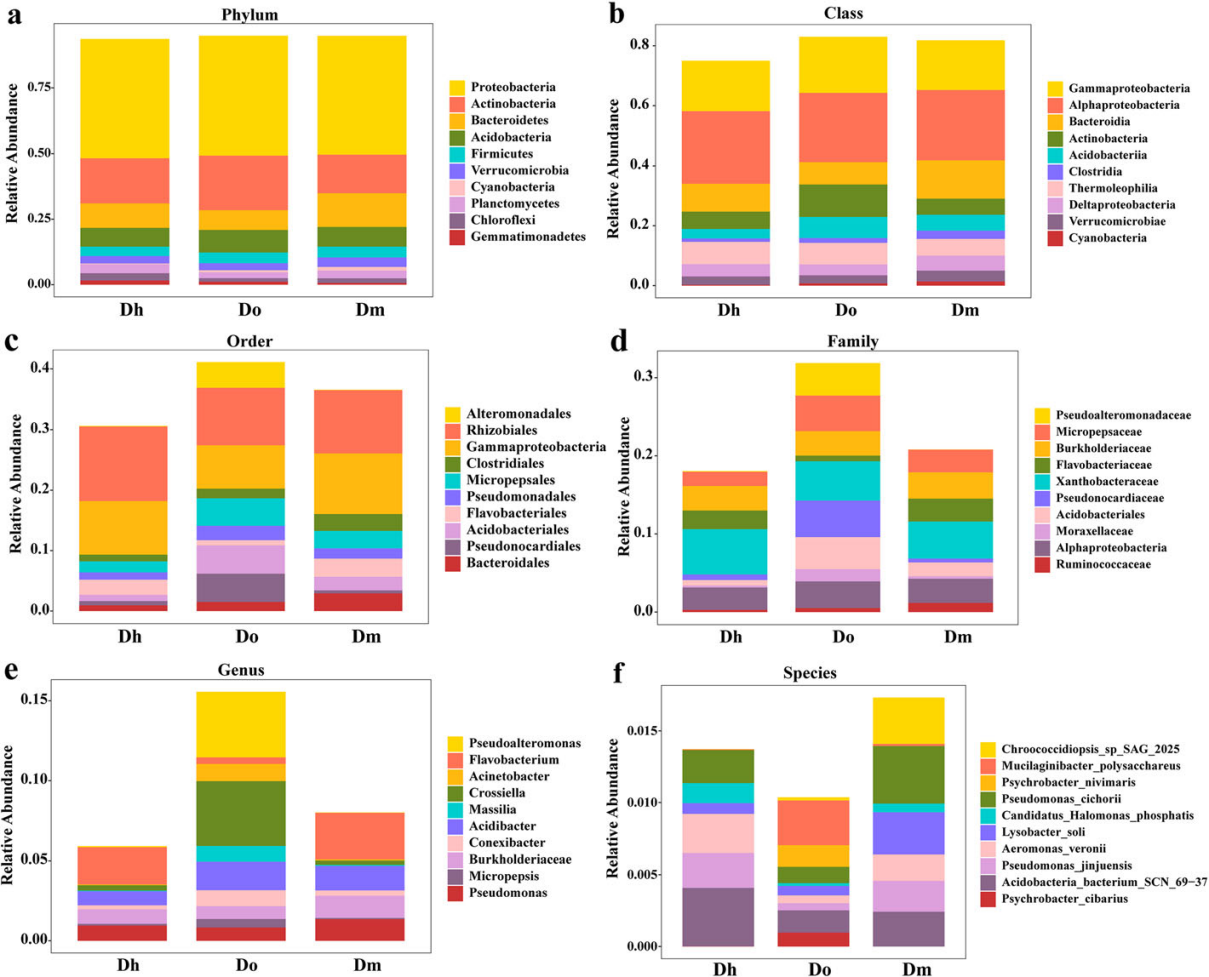
Top 10 relative abundances of bacterial communities classified at phylum (**a**), class (**b**), order (**c**), family (**d**), genus (**e**) and species (**f**) level in different *Dendrobium* species

Principal coordinate analysis (PCoA) was performed at the operational taxonomic unit (OTU) level (Fig. 2). The analysis of PCoA using the weighted UniFrac distance, indicated a distinct pattern in the rhizosphere bacterial communities associated with the two axes, explaining 29.58 and 19.62% of the total variation in the *Dendrobium* rhizosphere soil. Using the unweighted UniFrac distance, the two axes explained 17.64 and 11.79% of the total variation in the *Dendrobium* rhizosphere soil. Bacterial communities associated with the rhizosphere soil were clustered in three regions according to a PCoA using the unweighted UniFrac distance, corresponding to Dh, Do, and Dm. However, no clustering was detected using the weighted UniFrac distance from different *Dendrobium* species.

### Taxonomic classification and abundance

**Fig. 3:**
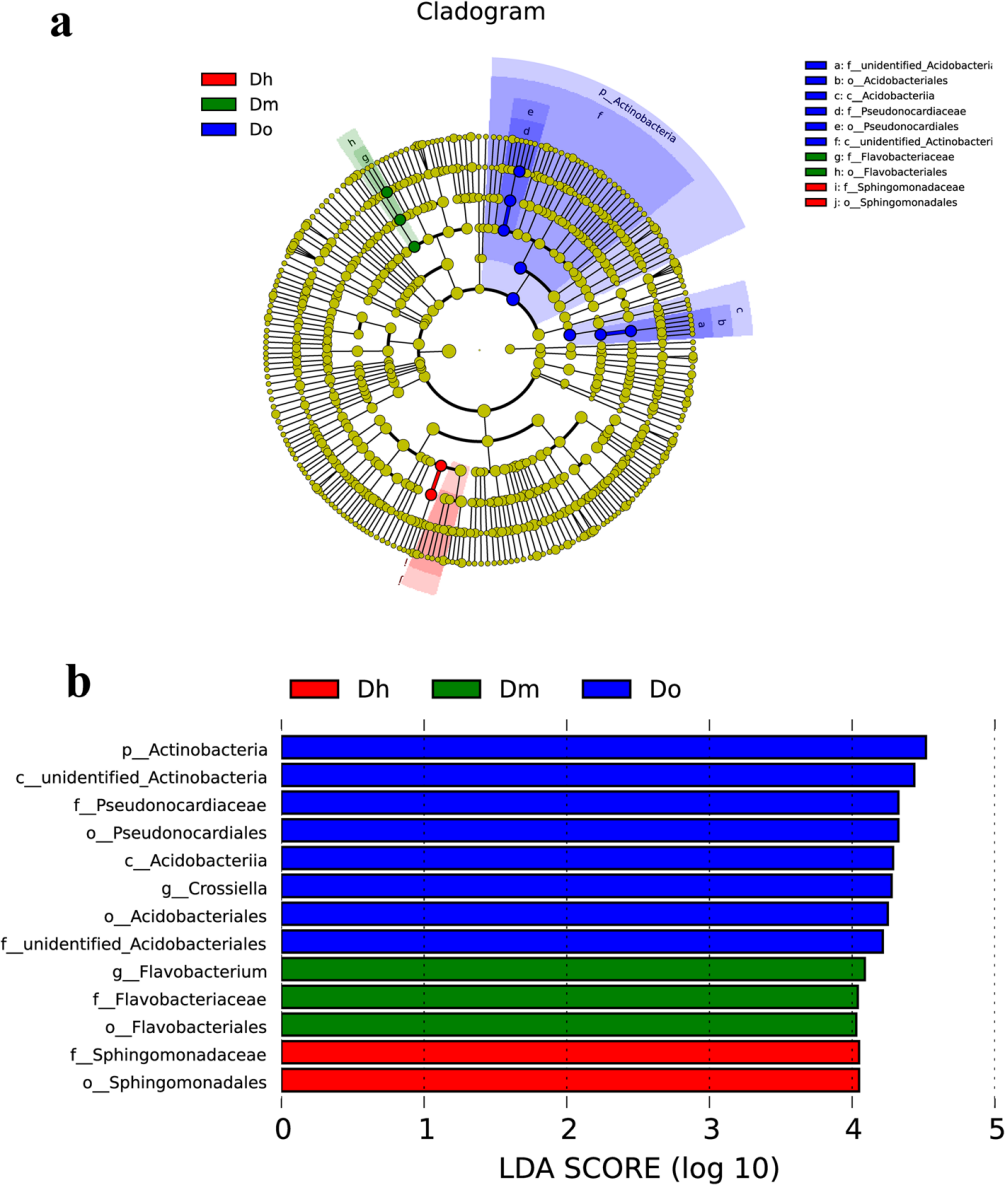
(LEfse) analysis of microbial abundance in Dh, Do and Dm. **a** Clustering diagram indicates phylogenetic distribution of the bacterial communities in three groups. **b** The histogram of LDA scores computed for differentially abundant microbe among different *Dendrobium* species identified with a threshold value of 4.0

Rarefaction curves for bacterial communities suggested that changes in OTU density within the different *Dendrobium* species was sufficiently captured, and the sequencing was relatively comprehensive in covering the microbial communities (Fig. 3a). After quality filtering and processing according to a 97% similarity, a total of 240,320 sequences were obtained from these three *Dendrobium* species, respectively: 80,101 for Dh, 80,109 for Do and 80,110 for Dm. The OTUs common to the different *Dendrobium* species are presented in Fig. 3b as a Venn diagram to assess the relationships among the bacterial communities. 5609 OTUs were shared by Dh, Do and Dm; 1041 OTUs were shared by Dm and Do; 926 OTUs were shared by Dh and Do; and 971 OTUs were shared by Dm and Dh. The numbers of OTUs unique to each species were as follows: 1007 for Dm, 909 for Do, and 716 for Dh. A total of 11,179 OTUs were detected in all samples (Fig. 3b).

**Fig. 4:**
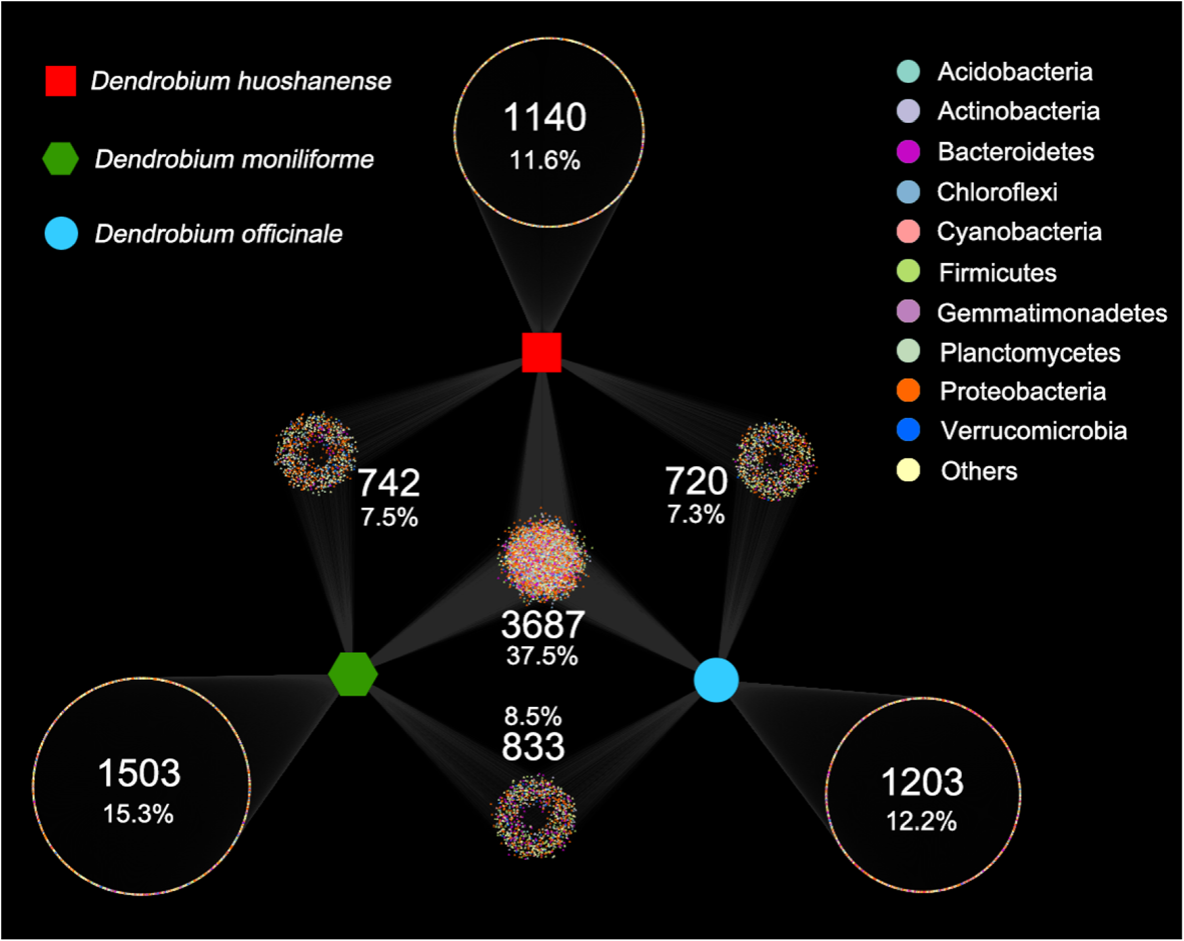
Bipartite association network showing positive associations between different rhizosphere soils and significantly associated phyla

According to the analysis of OTU annotation results, different *Dendrobium* rhizosphere soil bacteria include 2 kingdoms, 63 phyla, 72 classes, 159 orders, 309 families, 850 genera and 663 species (Fig. 4). Among all sequences, the dominant bacterial phyla (relative abundance > 1%) were *Proteobacteria*, *Actinobacteria*, *Bacteroidetes*, *Acidobacteria*, *Firmicutes*, *Verrucomicrobia*, *Planctomycetes*, *Chloroflexi*, and *Gemmatimonadetes*, with contributions of 45.41, 17.58, 9.94, 7.77, 3.93, 3.03, 2.88, 1.98 and 1.14%, respectively. The difference at the phylum level of different *Dendrobium* species is not large, but there were more and more obvious differences in the level of the class afterwards. At the order level, the relative abundance of *Alteromonadeles* on Dh and Dm was low, while the relative abundance on Do was relatively high. Pseudoalteromonadaceae at the family level and Pseudoalteromonas at the genus level also have the same relative abundance pattern. At the species level, although the top ten species of bacteria in the rhizosphere soils of the three *Dendrobium* species were the same, the relative expression abundances were very different.

### LEfSe analysis and co-occurrence patterns between rhizosphere soil microbiomes

**Fig. 5:**
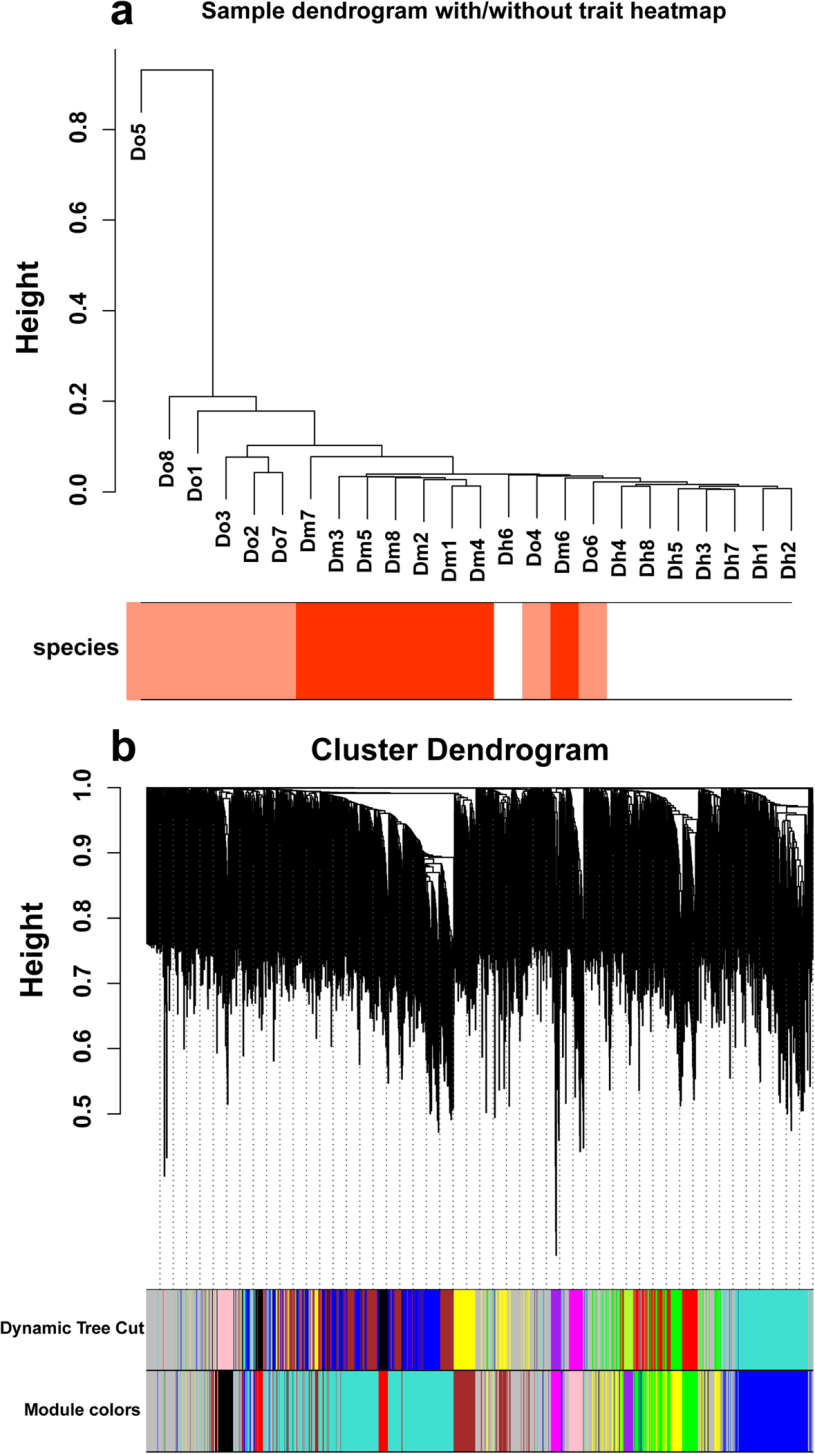
Clustering dendrogram. **a** Clustering dendrogram of 24 samples and heatmaps of species traits. The expression is from low to high, and the color transitions from white to red. **b** Clustering dendrogram of OTUs, with dissimilarity based on the topological overlap, together with assigned module colors. The clustered branches represent different modules, and each line represents one OTU

To further elucidate the possible interactions between identified bacterial dependencies in rhizosphere soil samples, linear discriminant analysis (LDA) and effect size (LEfSe) methods were used for quantitative analysis of biomarkers in different species. We detected significant differences in the abundance of bacterial biomarkers from different groups and identified a total of 13 biomarkers from all rhizosphere soil samples as shown in the branching diagram (Fig. 5b). The significant taxa in the Dh were affiliated with diverse phylogenetic groups, including the family Sphingomonadaceae, and order Sphingomoadales. In the Dm, the significantly abundant taxa were the genus Flavobacterium, the family Flavobacteriaceae and the order Flavobacteriales. In the Do, the significant taxa belonged to the phylum Actinobacteria, class unidentified Actinobacteria and Acdobacteriia, orders Pseudonocardiales and Acidobacteriales, family Pseudonocardiaceae and unidentified Acidobacteriales, and genus Crossiella, which were all abundant.

Network analysis is widely used in the exploration of complex microbial communities. In the rhizosphere soil of *Dendrobium*, the distributions and abundances of microbial genera are significantly different. In order to further explore the interaction between microorganisms in the root micro-ecological environment of *Dendrobium*, the contributions of different microbial populations to the overall community structure were evaluated using a bipartite association network to visualize the associations between the phylum and rhizospheric compartments (Fig. 7). In the bacterial community, 7 clusters of phyla were generated in the three kinds of *Dendrobium* species of the bipartite network. In the middle cluster of the bacterial community, 37.5% (3687) of the indicator phyla were associated with all the *Dendrobium* species, which is a large proportion of the indicator phyla.

### WGCNA analysis

#### Construction of gene co-expression network

We used the WGCNA program package in R software to construct the co-expression network and selected 11,179 OTUs from the sequencing results of the rhizosphere soil microbiome of 24 *Dendrobium* samples to construct the co-expression network. To begin with, we loaded the R package, read the expression value matrix, calculated and clustered the correlation coefficients of the expression levels of each sample, and drew a heat map of species characteristics. As shown in Fig. 5a, most of the samples are clustered into one group according to species. The relative abundance of OTU is ranked as *Dendrobium moniliforme* (Dm) > *Dendrobium officinale* (Do) > *Dendrobium huoshanense* (Dh). In order to make the co-expression network meet the condition of non-scale distribution, we needed a suitable weighting coefficient β; therefore, the function (pickSoftThreshold) was used to calculate β. While satisfying that the square of the correlation coefficient is close to 0.8, it was ensured that the average connectivity could not be too low. According to these two conditions, we chose β = 26, and the square of the correlation coefficient was 0.85.

The soft threshold obtained above was used to construct a scale-free network. Firstly, the adjacency matrix was calculated based on the expression value matrix, and then the topological overlap matrix reflecting the common expression similarity was deduced. Then, the topological overlap matrix was used for hierarchical clustering to draw a cluster tree that could jointly express the overall distribution characteristics of similarity. Finally, the dynamic shear tree algorithm was used to cut the resulting cluster tree. In this process, OTUs with high similarity in common expression were gathered into the same branch, and different branches of the cluster tree represented different modules, and each module was given a specified color (Fig. 5b). Ten co-expression modules were obtained after constructing the co-expression network (Table 1). Among them, turquoise module had the largest number of OTUs, which is 501, while purple module had the smallest number of OTUs, which was 29.

**Table 1:** The number of OTUs in 10 constructed modules

moduleColors	Freq
black	70
blue	268
brown	137
green	131
magenta	33
pink	43
purple	29
red	76
turquoise	501
yellow	134

#### Interaction analysis of co-expression module

**Fig. 6:**
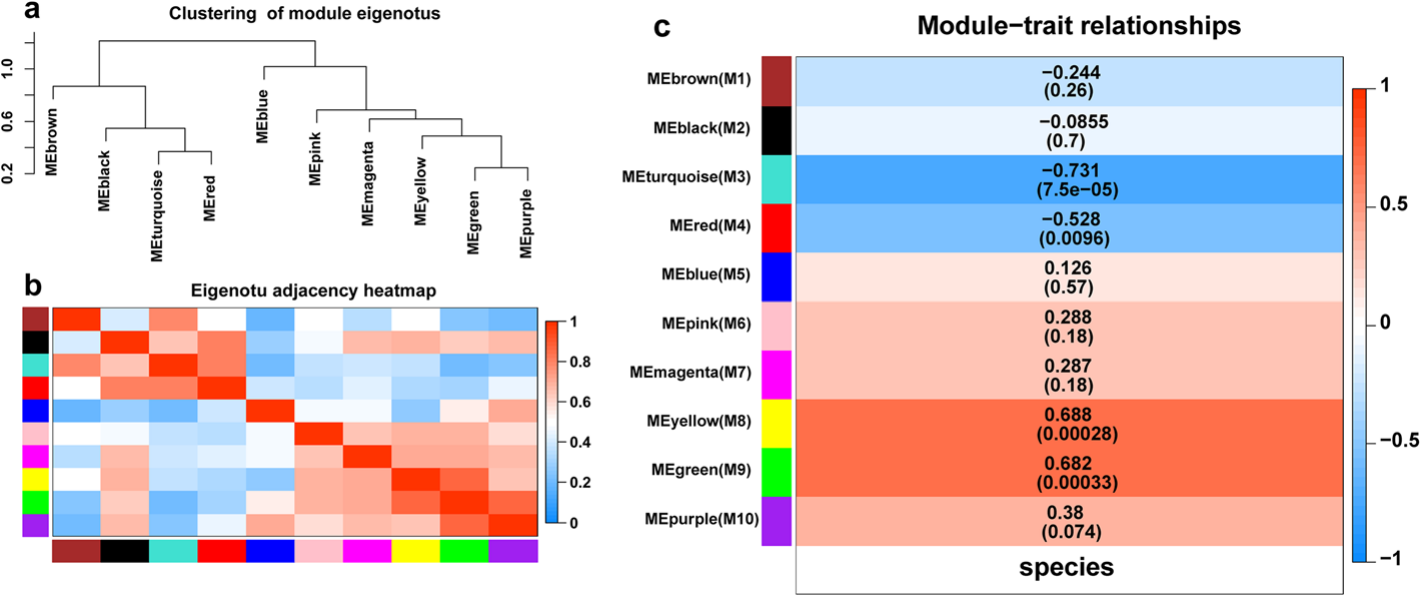
Analysis of connectivity of eigengenes in different module. **a** Cluster analysis of eigengenes. **b** The heatmap of connectivity of eigengenes. **c** Module-trait associations. Each row corresponds to a module characteristic gene (eigengene), and each column corresponds to a trait. Each cell contains a corresponding correlation and *p*-value. According to the color legend, the table is color-coded by correlation

In order to find the interaction relationship between co-expressed network modules, we analyzed the correlation of eigenvectors. Firstly, cluster analysis of eigenvectors was carried out (Fig. 6a). These 10 clusters were divided into two large clusters, including four modules (Brown, Black, turquoise and Red) and the other six modules. Therefore, the correlation between different modules was also significantly different, as shown in Fig. 6b. We analyzed the correlation between module feature vectors and species characteristics. As shown in Fig. 6c, four modules are highly correlated with species characteristics: turquoise module (*p*-value =7.5× 10^− 5^, correlation coefficient = − 0.731), Red module (*p-*value =0.0096, correlation coefficient = − 0.528), yellow module (*p-*value =0.00028, correlation coefficient = 0.688) and green module (*p-*value =0.00033, correlation coefficient = 0.682). Therefore, these four modules were further studied as modules of interest below.

#### Hub OTUs identification

**Fig. 7:**
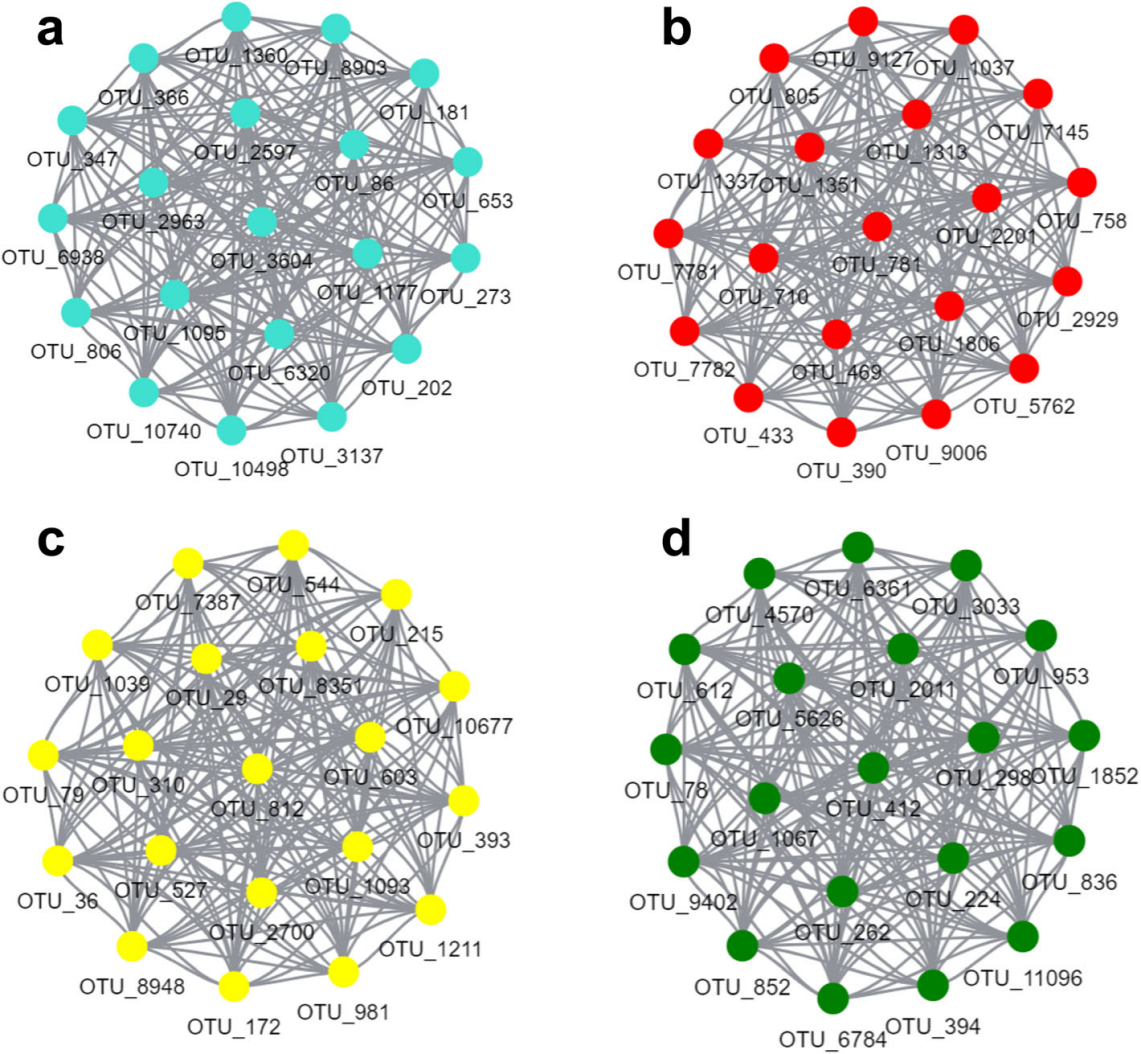
*Dendrobium* rhizosphere microbial co-expression network diagram. **a** Turquoise module microbial co-expression network diagram; **b** Red module microbial co-expression network diagram; **c** Yellow module microbial co-expression network diagram; **d** Green module microbial co-expression network diagram

From the above study, we obtained four modules with the most significant differences in species characteristics: turquoise, red, yellow and green. According to OTU notes (Additional file Table 1), we have carried out network analysis on these four modules (Fig. 7). In the turquoise module, there are 501 OTUs distributed in 19 phyla and 73 genera, among which the dominant bacteria phyla are Acidobacteria (13.2%), Actinobacteria (17.4%) and Proteobacteria (31.9%). In the red module, there are 76 OTUs distributed in 11 phyla and 22 genera, among which Proteobacteria (38.2%) and Actinobacteria (23.7%) are the dominant bacteria. In the yellow module, there are 134 OTUs distributed in 10 phyla and 34 genera, among which Proteobacteria (55.2%) is the dominant phylum. In the green module, there are 134 OTUs distributed in 11 phyla and 28 genera, among which the dominant bacteria phyla are Proteobacteria (51.9%) and Acidobacteria (19.8%).

## Discussion

Rhizosphere microorganisms can co-exist with plant roots, colonize and be maintained in roots, and play an important role in promoting plant growth and development [46]. Among these microorganisms, the utilization rate and sensitivity of bacteria to root exudates are far higher than that of fungi, and bacteria are the most active and dominant microorganisms in rhizosphere [47, 48]. In addition, the number and species of rhizosphere microorganisms have a direct impact on soil biochemical activity and nutrient transformation. Under the influence of various complex factors of natural conditions, there are great differences in rhizosphere microbial flora of different plants and even different genotypes of the same plant [49, 50]. So, in this study, bacterial communities associated with the rhizosphere of three different *Dendrobium* species were characterized by high-throughput sequencing during the maturity stage.

### Bacterial communities in rhizosphere soil of *Dendrobium*

It can be seen from the results that the dominant phyla in the rhizosphere soil of *Dendrobium* are Proteobacteria, Actinobacteria, Bacteroidetes, Acidobacteria, Firmicutes, Verrucomicrobia, Planctomycetes, Chloroflexi and Gemmatimonadetes. This indicates the richness and diversity of the rhizosphere microbial community of *Dendrobium*. However, the main dominant bacteria of *Dendrobium* are still concentrated in Proteobacteria, Actinobacteria and Bacteroidetes. Lundberg et al. found that the relative abundance of Proteobacteria, Bacteroidetes, Actinobacteria, Acidobacteria, Firmicutes and Gemmatimonadetes in Arabidopsis rhizosphere soil were relatively high, and were the dominant bacteria in Arabidopsis rhizosphere soil [8]. Ling et al. studied the rhizosphere microorganisms of watermelon, and found that Acidobacteria, Actinomycetes, Bacteroidetes, Cyanobacteria, Firmicutes and Proteobacteria were the main leading bacteria [51]. Davide et al. found that Actinobacteria, Bacteroidetes and Proteobacteria were dominant in barley rhizosphere soil [52]. Although there are differences in the bacterial communities of different plant rhizosphere soils, the dominant phyla of Actinobacteria, Bacteroidetes and Proteobacteria are the common dominant phyla of *Dendrobium* and the above-mentioned plants, indicating that these phyla may be the common dominant phyla of all plant rhizosphere bacterial communities. Proteobacteria predominates in all ecosystems, especially in soil systems [53, 54], due to the fact that Proteobacteria contains a large level of physiological, morphological and metabolic diversity, and that Proteobacteria is of great significance to the C and N cycles [55]. Proteobacteria reproduce fast, have good adaptability to unstable carbon sources, and are widely distributed in the global soil environment [56]. In general, the abundance of Proteobacteria or Acidobacteria in soil samples is the greatest. These bacterial groups rich in rhizosphere microorganisms of *Dendrobium*, are also found to be the dominant communities of other plant rhizosphere microbiomes [57, 58].

### Differences of bacterial communities in rhizosphere soils of three different *Dendrobium* species

According to the diversity indices alpha and beta, there were significant differences among the three *Dendrobium* species. The relative abundance of Acidobacteriales, Pseudonocardiaceae, Pseudoalteromonas and Pseudomonadales in *Dendrobium officinale* were higher than those in the other two species. Acidobacteriales is the dominant bacteria in the common plant rhizosphere bacterial community. Because Acidobacteriales can degrade complex root exudates such as cellulose and lignin, it plays a major role in the plant rhizosphere carbon cycle [59]. Pseudoalteromonas sp. secretes a variety of extracellular active substances, including proteins, polysaccharides, brominated compounds, extracellular enzymes, extracellular toxins, antibiotics and so on [60]. These substances have antibacterial, algicidal, bactericidal and cellulose degrading activities [61, 62]. Pseudomonadales is an important group of biocontrol microorganisms, and is also one of the most widely distributed microorganisms in nature. Its rapid reproduction, strong colonization ability and simple nutrition requirements have been widely studied for its inhibition of plant diseases and promotion of plant growth. In the existing studies, Pseudonocardiaceae is mainly associated with cellulose degradation and antibiotic synthesis [63–65]. It can be seen from the above that the function of the bacterial community in the rhizosphere of *Dendrobium officinale* may be stronger than that of the other two kinds of *Dendrobium*. In *Dendrobium moniliforme*, the relative abundance of Bacteroidetes is relatively high, and Bacteroidetes is a poor nutrient bacterium, which is suitable for growth in the environment with less absorbable nutrients such as organic matter and available nitrogen [66].

In bacterial species, we found that Lysobacter soli have a high relative abundance in *Dendrobium moniliforme*. This fungus has previously been isolated from the soil where ginseng is grown [67], and has been found in other plants to promote plant activity [68]. Psychrobacter ibarius has been found in *Dendrobium officinale*. Some researchers have isolated this bacterium from the root plane of *Angelica sinensis* [69]. This is a fungus related to polysaccharide synthesis. This is consistent with our previous research results [13]. Our previous research also found that among these three kinds of *Dendrobium*, the polysaccharides of two-year-old *Dendrobium* are higher than those in the other two kinds of two-year-old *Dendrobium*.

### WGCNA applied to the rhizosphere microbiome in *Dendrobium* species

In recent years, with the development of high-throughput sequencing technology, network analysis based on high-throughput omics data has become a popular big data processing method due to its high efficiency and convenience [70]. WGCNA, as one of the methods of network analysis, can specifically screen OTUs highly correlated with the target traits, divide them into multiple co-expression modules, and mine the core OTUs, which has been proved to be a fast, accurate and efficient method for data mining in potatoes and other plants [71, 72]. In order to understand the differences in the rhizosphere microbial communities of *Dendrobium*, this study used the WGCNA package in the R software to construct a co-expression network of the rhizosphere microbial community of *Dendrobium*, and explored the community structure of the rhizosphere microbes in a new way. A total of 10 modules were identified in the constructed *Dendrobium* rhizosphere microbial co-expression network, and the 4 modules with the highest correlation with species characteristics were further analyzed. Each module obtained 20 hub OTUs. By annotating these hub OTUs, the dominant bacteria phyla in the rhizosphere microbial community of Proteobacteria, Acidobacteria and Actinobacteria were finally obtained.

Compared with the results of this study, a total of 22 co-expression modules were identified in the rhizospheric co-expression network in the WGCNA analysis of rice root-associated microbiomes. By analyzing the OTUs annotations of the three microbial co-expression networks with the most significant differences among the three niches within the endosphere, rhizoplane and rhizosphere, it is concluded that the hub bacteria phyla in the rice rhizosphere microbial community is Proteobacteria, Actinobacteria and Bacteroidetes, which are highly similar to the dominant bacteria phyla we found in the rhizosphere microbial community of *Dendrobium*. The dominant microorganisms are relatively abundant in the soil and play an important role in the regulation of ecological functions [73]. Proteobacteria and Actinomycota are enriched in rhizosphere and non-rhizosphere environments, which is related to their strong adaptability. Among them, the Proteobacteria can survive and multiply in most environments and become the dominant bacteria because its outer membrane is mainly composed of lipopolysaccharide, which can protect its internal genetic material from external interference [74, 75]. In addition, the phylum Proteobacteria plays an important role in adapting to environmental changes and resisting adversity stress [76]. Actinomycota plays an important role in the decolorization of the toxic dye-triphenylmethane (TPM) in the soil [77], and plays a certain role in adaptation to arid environments [78]. Therefore, we speculate that the Proteobacteria and Actinomycota play a pivotal role in the plant rhizosphere microbial community, and they play an important regulatory role in the plant rhizosphere.

## Conclusions

Overall, the chemical properties of rhizosphere soil of *Dendrobium* and the climatic characteristics of *Dendrobium* cultivation were firstly analyzed. Then, combined with high-throughput sequencing technology, the rhizosphere soil microbial communities of different species of *Dendrobium* were studied, and the rhizosphere microorganisms and environmental driving factors of different species of *Dendrobium* were discussed. The main conclusions are as follows: the dominant bacteria in rhizosphere soil of *Dendrobium* are as follows: Proteobacteria, Bacteroidetes, Actinobacteria, and Acidobacteria. These are the dominant bacteria in the rhizosphere bacterial community. However, there are some differences in the bacterial communities among different *Dendrobium* species, and the smaller the bacterial level is, the greater the difference is. Through WGCNA analysis, it is found that in the rhizosphere microbial community network of *Dendrobium*, the hub flora is also in the Bacteroides, Acidobacteria and Proteobacteria.

## Supplementary Information

**Additional file 1: Table S1.** The taxonomy of OTU in different significant module.

## Data Availability

Raw amplicon sequence data related to this study were deposited in the NCBI Sequence Read Archive (NCBI SRA) under Bioprojects PRJNA638443.

## Abbreviations

AN: Ammonium nitrogen
AP: Available phosphorus
AK: Available potassium
OTUs: Operational taxonomic units
RDP: Ribosomal Database Project
PCoA: Principal coordinate analysis
